# Supplementary material for: A set of multi-entry identification keys to African frugivorous flies (Diptera, Tephritidae)
Source: Zookeys. 2014 Jul 24;(428):97–108. doi: 10.3897/zookeys.428.7366 (PMC4143993; doi:10.3897/zookeys.428.7366)
Supplement: Supplementary material 6 — Key to Ceratitis [file zookeys-428-097-s006.zip › SF6_ZooKeys_key to Ceratitis/key/SF6_ZooKeys_key to Ceratitis/Media/Html/Ceratitis lobata .htm]

Ceratitis (Pterandrus) lobata Munro


***Ceratitis*** **(*Pterandrus*) *lobata*
Munro**

*Ceratitis* *lobata* Munro, 1933b: 38.

Body
length: 4.20 (3.75-4.55) mm; wing length: 4.25 (3.80-4.50) mm.

Male

Head: Antenna yellow (sometimes yellowish orange). First
flagellomere 2-3 times as long as pedicel. Arista with short to moderately long
rays; basoventral rays shorter and sparser than basodorsal rays. Frons convex;
yellowish white to yellow, rarely with darker yellow patches near frontofacial
angle and in center; with short scattered setulae distinctly darker than frons.
Frontal setae usually well developed, sometimes ventrally more poorly
developed. Face yellowish white. Genal seta and setulae pale or reddish, latter
poorly developed. Occiput on dorsal part yellowish brown to brown colored,
except along dorsal margin.

Thorax:
Postpronotal lobe yellow, with brownish spot, sometimes not prominent. Scutal
pattern: ground color brown, occasionally black, microtrichose areas silvery
with ashgray shine, spots brownish black or black except poorly developed
sutural yellow spots, prescutellar yellow markings merged or separate. Scapular
setae dark. Scutellum yellowish white, basally with two merged dark spots,
apically with three merged� black spots, weakly incised, extending to basal
spots or almost so. Anepisternum on ventral half darker yellow; setulae pale.

Legs:
Yellow except where otherwise noted; setation typical for subgenus, mixed pale
and dark. Foreleg: coxa with tuft of silvery setulae ventrally; femur yellow
except dorsally on basal half black; anteroventrally with dense comb of silvery
setulae along entire length; posteriorly with poorly developed bush of long
palish setulae along entire length, posterodorsal setulae and row of dorsal
setulae on distal half longer, basally darker setulae, occasionally all setulae
more palish; ventral setae pale. Midleg: femur anteriorly with silvery oblique
line on distal 0.33, silvery shine conspicuous when viewed from certain angle;
ventrally with dispersed long pale setulae; midtibia ventrally with dense white
pilosity. Hindleg: femur at apical 0.25 with longer setulae dorsally and
ventrally. Midfemur and hindfemur anteriorly with brownish patch on
ventral half.

Wing:
Pattern; bands yellow and brown; with incised loba on ventral apical part.
Marginal band forming continuous band with anterior part of discal band;
cubital band free; medial band absent; crossvein R-M proximal to middle of
discal cell. Apex of vein R_1_ distal to level of crossvein R-M.
Crossvein DM-Cu oblique anterobasally.

Abdomen:
Mostly yellow. Tergite 1 yellow with brown patches posteriorly; tergites 2 and
4 with silvery transverse band along� posterior half; tergite 3 posterior half
brown; tergite 5 yellow with brown patches laterally; sometimes abdominal
tergites generally with darker brown appearance. Setation and banding typical
for subgenus. Male epandrium in lateral view distinctly broadened; posterior
lobe of lateral surstylus with base broadened, apical end sharply pointed.

Female

As
male except for the following characters: Frons yellow, darker patches more
distinct. Slight darker coloration along gena. Genal seta and setulae dark and
well developed. Ventral half of anepisternum brown, with dark setulae
posteriorly. Legs without feathering, dark yellow, femora more orange-brownish;
pilosity and ventral setae of forefemur dark. Wing banding usually darker than
in male; without incised lobe. Abdomen sometimes more extensively brownish
colored. Oviscape shorter than preabdomen. Aculeus at least eight times longer
than wide; tip in lateral view ventrally curved, pointed, and with lateral
margin straight.

(Description
after De Meyer & Freidberg, 2006)
